# Data on the agricultural household's dietary diversity and health in the South West geopolitical zone of Nigeria

**DOI:** 10.1016/j.dib.2020.105413

**Published:** 2020-03-10

**Authors:** Abiodun Olusola Omotayo

**Affiliations:** Food Security and Safety Niche Area, Faculty of Natural and Agricultural Sciences, North West University, Private Mail Bag X2046, Mmabatho 2790, North West Province, South Africa

**Keywords:** Body mass index, Food intake, Health outcome, Nutrition, Rural development

## Abstract

Food intake remains an essential component of human health life and productivity. Poor health inextricably threaten the ability of several developing nations to achieve the Millennium Development Goals by 2015, this stubborn threat is still a major concern to the achievement of the sustainable development goals (SDG. 2030). The economic burdens of poor nutrition and ill health in the development of African continent cannot be overemphasized. Therefore, eating a varied, well-balanced food groups daily, in the recommended amounts is important. Considering the existing malnutrition and ill health situation report in Nigeria, rural farmer's dietary diversity and health record is important for pertinent policy evaluation since these people are the principal operators of the nations' food system but yet one of the most vulnerable category of the countrie's working class. The survey that gave this dataset was conducted through a multi stage sampling technique with a well structured questionnaires with in the months of September 2014 and April 2015 from households selected from 18 randomly sampled villages. The administered questionnaires were divided in seven sections namely; respondent's socio-economic characteristics, health and environmental profile, food utilization and nutrition, requested information about respondent's agricultural labour productivity, agricultural production cost and return, cost implication of health and nutrition and dietary diversity nutrition and other problems. The questionnaires were written in English language but translated in local language during the interview for ease of understanding by the participants, the survey successfully ended with a total of 420 properly filled and captured questionaires which was quite representative. The dataset is hereby made available as it is considered vital for policy recommendations.

Specifications table

**Table:** 

Subject	Agricultural sciences
Specific subject area	Food value chain, food intake and health
Type of data	Table, chart, figure and SPSS data file
How data were acquired	Rural household's survey with a well-structured questionnaire (Submitted with the article)
Data format	Raw and analysed
Parameters for data collection	Face to face interviews
Description of data collection	A survey that gave this dataset which was conducted with a well structured questionnaires administered in 18 randomly sampled villages, making a total of 420 rural interviewed households
Data source location	South West geopolitical zone of Nigeria
Data accessibility	Submitted with the article

## Value of the data

• The data provides information on dietary diversity of farmers and health capital.
• The data present descriptive representation of socioeconomic characteristics of the households as it relate to their nutritional diversity and their health outcome. This is geared towards understanding the dietary diversity and health outcome of the rural farming households.
• This data provides valuable information that may be functional at different levels for both government organizations (GOs) and non-government organizations (NGOs) in order to formulate appropriate policy and intervention strategy for the improvement of rural food system and health.
• The data can be further analysed to understand the synergy between dietary diversity and health. It can also explore some correlates of dietary diversity and health of farmers.
• The dataset can assist to promote the understanding of the factors explaining food diversity, health and farming household's productivity.

## 1 Data description

The dataset was compiled after a questionnaire administration, coding and data cleaning of data from 420 farming households between September 2014 and April 2015. Table 1 reveals that majority of the respondent fall into the age intervals of 40–60 years with 58.90%, 54.20% and 54.20% in Oyo, Ogun and Osun states, respectively. Also, the average age of households’ head across the selected states and their standard deviation (in parenthesis) were 54.6 years (11.30), 51.0 years (11.840) and 53.8 years (11.18) in Oyo, Ogun and Osun states, respectively [1].

**Table 1:** Respondents distribution according to age across the selected states.

Age	Oyo State		Ogun State		Osun State		Study Area	
	Freq	%	Freq	%	Freq	%	Freq	%
21–40	26	14.40	28	23.30	19	15.80	73	17.38
41–60	106	58.90	65	54.20	65	54.20	236	56.19
61–80	48	26.70	27	22.50	36	30.00	111	26.43
Total	180	100	120	100	120	100	420	100
	*X*=54.6	SD=11.30	*X* = 51.0	SD=11.84	*X*=53.8	SD=11.18	*X=53*	SD=11.44

*Note:* SD= Standard deviation; x=Mean; %= Percentage; Freq=Frequency.

Table 2 shows that 82.20%, 80.80% and 80.00% of the respondents were male in Oyo, Ogun, and Osun respectively.

**Table 2:** Sex distribution of respondents across the selected states.

Gender	Oyo State		Ogun State		Osun State		Study Area	
	Freq	%	Freq	%	Freq	%	Freq	%
Male	148	82.20	97	80.80	96	80.00	341	81.19
Female	32	17.80	23	19.20	24	20.00	79	18.81
Total	180	100	120	100	120	100	420	100

### 1.1 Distribution of respondents according to marital status

Table 3 shows that majority of the respondents representing 77.20%, 68.30%, and 76.70% are married in Oyo,Ogun and Osun states respectively while 12.20%, 19.20% and 10.80% of the respondents are single [2].

**Table 3:** Distribution of respondents by their marital status in the selected states.

Marital Status	Oyo State		Ogun State		Osun State		Study Area	
	Freq	%	Freq	%	Freq	%	Freq	%
Singles	22	12.20	23	19.20	13	10.80	58	13.81
Married	139	77.20	82	68.30	92	76.70	313	74.52
Divorced	11	6.10	4	3.30	4	3.30	19	4.52
Widow(er)	7	3.90	9	7.50	10	8.30	26	6.19
Separated	1	0.60	2	1.70	1	0.80	4	0.95
Total	180	100	120	100	120	100	420	100

### 1.2 Households’ dietary diversity score across the selected states

**Fig. 1:**
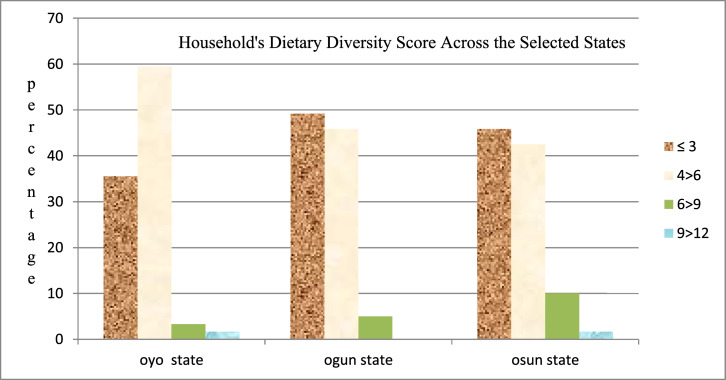
Respondents households HDDS across the selected states.

Fig. 1 shows the dietary diversity scores which was based on the 12 food groups set by the FAO. The mean food intake score recorded across the selected states were 5.20, 5.10 and 4.31 from Oyo, Ogun and Osun states as against the mean cut-off point of 6 recommendation.

### 1.3 Body Mass Index (BMI)

This measures the variation of nutrition and health status in the human life cycle. It is a very good indicator of health at individual and population level. It also serves as a proxy measure of adiposity, which is independent of gender, age and ethnicity. The various categories are as tabulated in Table 4 below:

**Table 4:** BMI classifications are as follows.

BMI Figure	Categories
< 18.50	Underweight
18.50–24.90	Normal/desirable weight or healthy
25.00–29.90	Overweight
30.00–34.90	Obese I
35.00–39.90	Obese II
>40.00	Severely Obese

**Fig. 2:**
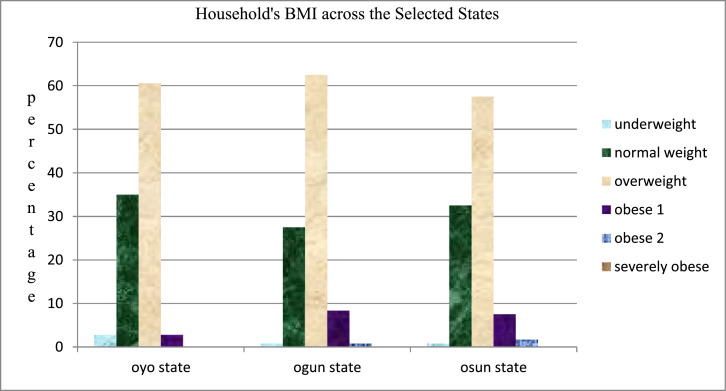
Distribution of respondents households BMI across the selected states.

Fig. 2 reveals the body mass index of the farmers with a minimum value of 15 kg/m², maximum BMI of 39 kg/m² and average BMI of 26.08 kg/m² ± 2.88. Majority (60.24%) of the farmers were overweight as compared with the other groups of 1.17%, 32.14%, 5.24 and 0.71 being underweight, healthy, obese 1 and obese 2 respectively. However, when the body mass index (BMI) was further analysed across the selected states of the study area. This indicates an average BMI of 25.63, 26.42 and 26.22 kg/m² for Oyo, Ogun and Osun states respectively.

## 2 Experimental design, materials, and methods

The survey and data collection that leads into the compilation of this dataset was conducted between September 2014 and April 2015. A multi-stage sampling procedure was adopted in the selection of respondents in the study from Ogun, Oyo, and Osun state. Data were purposively selected from the six states in the geopolitical zone, based on the prominence of agricultural activities in these states. The major occupation of the people of this geopolitical zone include farming, artisans and agricultural products’ processors and marketers. The three selected states were purposefully chosen because they were popular with small scale agricultural farming and are the food hub of the geopolitical zone. Data was collected through a well structured questionnaire (with a pass reliability test using a split half technique to determine the reliability of the instrument which gave a high-reliability coefficient of *r*=0.81, indicating that the instrument was consistent and highly reliable) which includes the participants’ demographics characteristics, housing conditions, environment-related issues, consumption expenditures pattern, cost and food compositions, cost and returns of enterprises, nutrition, and health status. The utilised questions were translated into the local language of the respondents during administration and their response was recorded in English language. The second stage was the selection of one Agricultural Development Programme (ADP) zone from each selected state regarded as the food basket of the state. The third stage was a random selection of two (2) Local Government Area in each of the ADP zones. Based on the total household population figure provided by the National Population Commission of 203,631 for the six (6) selected LGA, four hundred and fifty (450) households were then randomly selected from 18 villages (3 prominent villages from each LGA) using a proportionate properly filled sample of 130, 160, and 130 from Ogun, Oyo and Osun respectively making a finally compilled 420 households dataset. This was robust and representative of the selected households in the research.
